# Superior effect of forceful compared with standard traction mobilizations in hip disability?

**DOI:** 10.1080/14038190701395739

**Published:** 2007-08-24

**Authors:** Kjartan Vaarbakken, Anne Elisabeth Ljunggren

**Affiliations:** Section for Physiotherapy Science, Department of Public Health and Primary Health Care, Faculty of Medicine, University of Bergen, Kalfarveien 31, 5018 Bergen, Norway

**Keywords:** Hip disability, randomized controlled trial, treatment outcome

## Abstract

The objective of this study was to compare the effectiveness of two compiled physiotherapy programs: one including forceful traction mobilizations, the other including traction with unknown force, in patients with hip disability according to ICF (the International Classification of Functioning, Disability and Health, 2001; WHO), using a block randomized, controlled trial with two parallel treatment groups in a regular private outpatient physiotherapy practice. In the experimental group (E; *n* = 10) and control group (C; *n* = 9), the mean (±SD) age for all participants was 59 ± 12 years. They were recruited from outpatient physiotherapy clinics, had persistent pain located at the hip joint for >8 weeks and hip hypomobility. Both groups received exercise, information and manual traction mobilization. In E, the traction force was progressed to 800 N, whereas in C it was unknown. Major outcome measure was the median total change score ≥20 points or ≥50% of the disease- and joint-specific Hip disability and Osteoarthritis Outcome Score (HOOS), compiled of Pain, Stiffness, Function and Hip-related quality of life (ranging 0–100). The mean (range) treatments received were 13 (7–16) over 5–12 weeks and 20 (18–24) over 12 weeks for E and C, respectively. The experimental group showed superior clinical post-treatment effect on HOOS (≥20 points), in six of 10 participants compared with none of nine in the control group (*p* = 0.011). The effect size was 1.1. The results suggest that a compiled physiotherapy program including forceful traction mobilizations are short-term effective in reducing self-rated hip disability in primary healthcare. The long-term effect is to be documented.

## Introduction

This article is about treatment of one group of patients earlier characterized into two different groups, namely (i) hip disability and (ii) hip osteoarthritis (OA). Disability serves as an umbrella term for impairment, activity limitation and participation restriction in the ICF (1). Patients with hip disability have impairments that include pain, stiffness and decreased joint mobility as part of the health domain Body Functions (1). We found no prevalence data on hip disability in PubMed, EMBASE or AMED, but in Sweden in 2004, the prevalence of self-reported hip disorders was 32% and increased with age from 18% among males from 38 to 47 years to 42% among females from 48 to 67 years (2).

Also in Sweden, the age-specific prevalence of X-ray verified hip OA is shown to fit an exponential curve for which it increased from below 1% in the age group <55 years to 10% in the age group >85 years (3). Specified, the prevalence of self-reported hip OA in the Netherlands, defined as patients told by their general practitioner as having this disease, is about twice of that for X-ray verified hip OA (4). The same study showed about half of those with the disease to receive regular medication treatment.

In sum, patients in the combined group “hip OA and hip disability” present a major health concern.

Hip disability is a closely related clinical category to hip OA. The only difference in primary physician-set diagnostics seems to be if there are joint space narrowing (JSN) over 2.5 mm on X-ray pictures (4). This differentiation seems odd, since most clinical signs and symptoms have been found to be unrelated to this degree of radiographic change (5). Further, the validity of the much used American College of Rheumatology's criteria for classifying hip OA (6,7) has been challenged, especially regarding direction of limited range of motion (ROM) (8,9). Because the two concepts overlap so strongly and because physiotherapists intervene with the same approach whether the patients have X-ray findings or not, we choose to present epidemiology and treatment effect data for both concepts in this introduction.

Clinically, hypomobility in individuals with painful OA hips has sparsely been shown reversible by exercise therapy (10–13), but recently, in a randomized controlled trial (RCT), manipulations by high-velocity small amplitude rotational thrust during traction combined with self-stretching were reported to improve ROM and disability better than exercise therapy (14).

Joint mobilizations, defined as passive joint movement with rhythm and grade such that the patient can resist it (15), have until now shown no therapeutic effect in patients with hip OA (16–19). Traction as passive mobilization of 100–250 N has documented negligible treatment effects in two RCTs on ROM, pain, stiffness and function in individuals with hip OA (16,17). Still, this treatment in earlier textbooks was claimed to be highly effective for hip pain (15,20,21). The discrepancy between the research evidence and clinicians' claimed experience might be due to non-adequate force-progression treating this massive joint in prior trials. Traction forces of at least 400–600 N have been shown necessary to deform the capsule into the linear region of the load–deformation curve in nine of 12 healthy persons (21,22). Three persons even needed higher forces. Although these data were reported 16 years ago, they seem neither to have reached the Norwegian manual therapy textbook (23) nor Norwegian physiotherapy schools, according to our knowledge.

We therefore undertook an RCT to compare the effect of a compiled physiotherapy program including manual traction mobilization graded up to 800 N (21) with a compiled physiotherapy program including traction mobilizations of unknown forces (23). The latter is standard praxis being taught in Norwegian physiotherapy schools for patients with hip disability and hip OA. The upper force limit was set due to our hypothesis that such traction forces would deform the stiff hip joint capsule into the linear region of the load–deformation curve. Thus, our treatment hypothesis was that patients who receive a compiled physiotherapy treatment including mobilization forces up to 800 N will experience superior important clinical effects as compared with those who receive a compiled physiotherapy treatment with unknown traction forces.

## Materials and methods

### Study design

An RCT with two parallel treatment groups was carried out. The treatment sequence was generated by one of the authors (KV) using a block partition method by a randomly numbered table (24). The allocation concealment was realized by numbered tickets in opaque envelopes sealed and shuffled into an envelope containing one block sequence. The block sizes were decided by the flip of a coin between four and six. The total sequence was generated in advance of patient enrolment for a total target sample of 50 participants based on a power estimate of 80%, using a nomogram (25), where the standardized treatment difference was set to 0.80 for the primary outcome and the α level to 0.05.

During enrolment, patients underwent a clinical test procedure performed by KV. After signing an informed consent, patients chose their own envelope, signed it before opening and then signed the allocation list. No efforts were made regarding blinding of the therapists or patients.

### Subjects

Candidates were men and women between 30 and 90 years referred to outpatient physiotherapy for hip disability in Oslo County, Norway, from December 2003 to October 2004, who had: (i) persistent pain in or from the hip daily in the last 8 weeks (26), (ii) reduced hip mobility, defined as passive ROM less than two standard deviations (SD) of the reported mean active ROM for their age group in at least one direction on the painful side (27), and (iii) pain located toward the hip joint when tested by passive firm end-pressure in orthogonal plane movements. Point (ii) was estimated by KV during the clinical examination, while (iii) was ensured by the patients pointing directly at the inguinal crease as the tests were performed.

Patients were excluded if they had: (i) history or signs in accordance with labral injury and, or a free intra-articular body, (ii) trauma, deformity or OA due to early hip disease, (iii) medically diagnosed inflammatory disease, (iv) showed obvious neurological signs such as sensory or motor paralysis, (v) other diseases, which entailed a powerful constraint on the physical, psychological or social functioning, (vi) additional pain from the lower back, pelvis, knee and/or ankle, which overshadowed pain from the hip, (vii) problems receiving information due to inadequate hearing, sight, intellect or knowledge in the Norwegian language, (viii) fulfilled criteria for total hip replacement (28). All physiotherapists (*n* = 556) and primary physicians (*n* = 441) reported in lists from the National Health Service to work in Oslo County received a written invitation to refer patients to the project. This gave very few volunteers, and nearly all participants were recruited by KV from the waiting lists of four physiotherapy clinics. General practitioners referred two patients.

**Figure 1:**
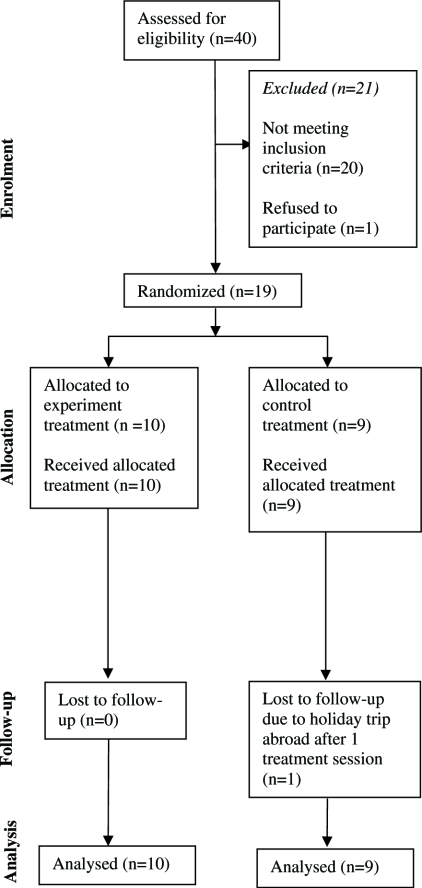
The progress of the participants through the trial phases. Not meeting inclusion criteria due to: lumbar pain (*n* = 6), pelvic pain (*n* = 4), enthesopathies without joint pain (*n* = 7), too small ROM deficits (*n* = 3). The one dropout was given the median change score for his group, implementing the intention-to-treat analysis, which explains why there was data for nine participants being analyzed in the control group.

Point (vi) was evaluated by repetitive stress tests directed sequentially to the lower back, pelvis and hip joint. The patients were simultaneously urged to express which test was most provocative and whether it reproduced their usual pain. They were asked to locate the pain using one finger. KV based his evaluation on the patients' expressions held together with information of which joints received the main joint stress at the given point of expression. The pelvic joint was stressed directly according to Hesch (29). The lumbar spine was stressed through neurodynamic tests (30) and by direct posteroanterior springing tests to the segments while lying prone. KV added countertorques to confine the joint reaction forces in the joints being tested. For instance, when stressing the hip joint, contertorques around the sacroiliac axis were added to the torques caused by movement of the femur. One practical example: with the patient lying prone, posteroanterior pressure was added to the tuber ischii while extending the hip. In all tests, the force progression principle (31) was used, starting at the painless side or level. Each clinical examination took approximately 2 h, the anamnesis included. A flow diagram of the progress of the trial is shown in Figure 1.

### Interventions

In the experimental group, all treatment was performed in one clinic by two physiotherapists who had over 10 years of experience with this method (21) and this group of patients. The last month before the trial, the therapists once daily calibrated their force effort during traction by applying forces to a model of a foot connected to a hanging scale, which again was connected to the bench. This was also done once weekly during the trial. When blindfolded, the therapists applied forces within an accuracy of 50 N in the trial period.

**Figure 2:**
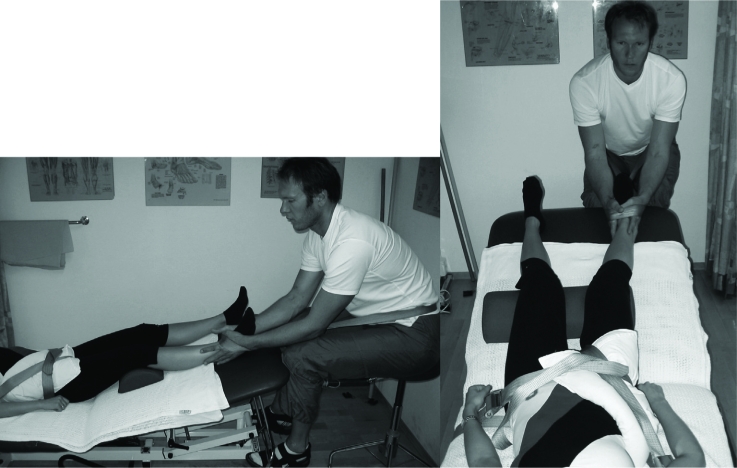
Physiotherapist mobilizing in traction on the patient's right hip. The pillow bolsters the pubic and the anterior superior iliac spines. The belt resisting lateral pelvis glide loops the metal under the patients left side of the plinth, and turns around the pelvis in a level directly inferior to the two anterior superior iliac spines to reconnect. Pelvis caudal glide is resisted by a belt looped from under the superior right-hand side of the plinth, turn around the ipsilateral pubic bone to recouple.

The mobilization technique of Samuelsen & Høiseth (21) was carried out with the patient lying supine on the left-hand side of the plinth (while treating the right side), at first with the hip in the maximal loosely packed position (23), which has been shown to facilitate joint separation (22). When joint stiffness in this position decreased, as judged by the therapist, traction was performed with the joint pre-positioned in the hypomobile direction (Figure 2). Each patient received about 15 min of manual traction mobilization in each session, graded according to Maitland (15). The average holding time in the first sessions varied from 20 to 40 s, and decreased to 10–15 s as the therapists judged improvement of the joint's elasticity.

The therapists in the experimental group also used deep soft-tissue techniques (32), strength exercises, and self-stretching (33,34) – targeting trigger points, weak muscles and stiff muscles, respectively. One strength exercise has been specially designed to target the m. quadriceps coxae – comprising mm. piriformis, gemellii and obturator internus: The patient horizontally abducts the 90° flexed hip joint lying on his/her side or in the so-called “three extremity standing” position. The lever arm is adapted by changing the knee angle or the patient's position in the gravity field. Furthermore, the therapist stretch the same joint near muscle group by combining hip flexion, adduction and external rotation, as this has been shown in cadavers to lengthen the muscles more than doing the intuitive hip internal rotation in combination with the two other motions (35). The information (28), given in a pamphlet, encouraged among other things taking out full ROM daily, sitting for a maximum of 20 min continuously without movement and doing varied low-impact activities regularly.

In the control group, the participants were treated in six clinics by eight physiotherapists, of whom three were licensed specialists in manipulative therapy. The therapists had used the standard manual traction mobilization technique (23) on a regular basis, and had at least 5 years of clinical experience. Mobilizations (23) were performed without standardization of applied forces, and therapists were urged to perform treatment as normal. The patients received information, exercises and soft-tissue techniques governed individually by each therapist. KV asked for the therapists' clinical experience, on the telephone before the trial, and received treatment reports for all but one patient after the trial. The information lacking was obtained by telephone.

In both groups, patients with bilateral problems received treatment on both sides, giving a maximum of 10 min of mobilization for each hip. Patients received soft-tissue techniques, exercises and information for which no restrictions were imposed by the trial administrator. Other treatment modalities were discouraged during the treatment period. No effort was made to control compliance regarding home exercises. The trial protocol stated two treatment sessions per week over 12 weeks in both groups (see results). Each treatment session lasted 30 minutes in total.

One patient in the control group received a co-intervention of therapeutic low-intensity ultrasound. No participants in either group withdrew because of increased complaints, nor received therapy from other health professionals. Neither was there reported any adverse effects.

### Procedures

Before the enrolment, all participants completed a questionnaire regarding demographic variables, previous complaint(s), duration of symptoms, co-interventions and previous treatment with manual traction mobilization. The patients were recorded to have radiographic hip OA when showing positive X-ray reports and stating to have received hip OA diagnoses orally from their general practitioners. The use of non-steroidal anti-inflammatory drugs (NSAIDs) and analgesics were permitted as needed in both groups and were recorded at baseline (week 0) and at follow-up (week 12). The self-rating questionnaires were filled in at home after the clinical examination. The patients with bilateral hip disability were encouraged to refer to their general situation considering both hips when reporting pain and stiffness.

### Outcome assessment

The main outcome variable was the median total change score of the patient self-reporting questionnaire Hip disability and Osteoarthritis Outcome Score (HOOS) (36), as stated in the trial protocol to the Regional Ethics Committee (for cut-off values, see statistics). The HOOS comprises five subscales and 39 items; each item has five alternatives scored from 0 to 4. The total median HOOS (HOOSt) was calculated adding the five 0–100 scaled subscales and dividing them by 5. The subscales of HOOS represent secondary outcome variables (Table I).

**Table I:** Baseline characteristics of all participants (*n* = 19) including the total Hip disability and Osteoarthritis Outcome Score (HOOS) and subscales in medians and interquartile range (IQR), if not otherwise specified.

Variables	Experimental group (*n* = 10)	Control group (*n* = 9)
Demographics		
Age, years, mean (SD)	62 (14)	57 (21)
Body mass index, kg/m^2^, mean (SD)	24 (4)	25 (7)
Gender, *n*, females	6	2
Prognostic characteristics		
Duration of complaints, years, mean (SD)	10 (6)	5 (9)
Distal spread of pain, *n*		
Nates, thigh, calf, foot	1, 2, 2, 5	1, 5, 1, 2
Hip pain, *n*		
Uni, bilateral	4, 6	6, 3
Hard physical work which aggravates condition	4	4
OA, X-ray verified, *n*	8	7
HOOS		
Stiffness^a^	43 (21)	55 (25)
Pain	46 (28)	44 (19)
ADL^b^	38 (28)	41 (19)
R&S^c^	63 (31)	56 (22)
HR-QL^d^	59 (16)	63 (34)
Total HOOS^e^	48 (17)	53 (23)

a Symptoms other, included stiffness;

b Activity limitation in daily living;

c Activity limitation in recreation and sports;

d Hip-related quality of life;

e Total Hip disability and Osteoarthritis Outcome Score HOOS = [(Σ5HOOS subscores)/5], Scores: 0 (no disability)–100 (worst possible disability). SD, standard deviation.

The Swedish version LK1.1 of the HOOS has been validated for patients with hip disability and hip OA (36,37), and it was translated into Norwegian through an ethnocentric approach (38). The scale was first translated from Swedish to Norwegian by the second author (EL), who is native Norwegian with a thorough knowledge of the Swedish language. The EL-version was then translated back to Swedish by OB (see acknowledgement), a Swedish Master of Science colleague, who at that time had lived for 5 years in Norway. The three different versions – the original LK1.1 and the two translations – were then compared by KV. Two items were dissimilar in the two Swedish versions. The developer of the scale (39) was consulted, before KV and the two translators discussed the items and reached a consensus. The Norwegian version is now electronically available (40) and is currently being further evaluated at our university.

Secondary outcome measurements of passive ROM were taken using a goniometer with a scale marked in 5° increments as prior validated (41). The two raters, 3rd-year physiotherapy students, were trained in a protocol adapted from Norkin & White (42). We adapted the rotations to be taken with the patient sitting instead of prone. This skill acquisition was guided by KV for 9 h in the month before baseline. The protocol was tested and found unreliable according to the requested minimal clinical important difference of 5° in each direction. The test–retest procedure, results and discussion have been reported earlier (43). Because of the inadequate reliability of the tests, the ROM results are not presented in this article.

### Data analysis

Analysis was performed according to the intention-to-treat principle (44). The only patient who dropped out was given the median change scores for the rest of the control group to which he belonged (Table I).

A null hypothesis of no clinical difference in HOOS between the two treatment groups was expressed against the alternative hypothesis that the experimental group would gain superior clinical improvement. Descriptive measurements of the change scores within and between groups were in medians, interqartile ranges (IQR) and percentages. The differences between the groups were tested by the Mann–Whitney *U*-test, presented with *p*-values and non-parametric confidence intervals (CI) (45). Specified, the non-parametric 95% CIs were calculated by:

$$K=W_{\alpha /2}-\frac{n(n-1)}{2}$$

where W_α/2_ is 100α/2 percentile of the distribution of the Mann–Whitney test statistics. The *K*th smallest to the *K*th largest of the *n* × *m* differences then determine the 100(1 − α)% CI. Values of *K* for finding approximate 95% CI were taken from a table according to the size of *n* (=number of patients in the experimental group) and *m* (=number of patients in the control group).

The group differences in proportions were tested for significance by Fisher's Exact Test. Cutpoints for clinical improvement were set to a change of ≥20 points (absolute criteria) and ≥50% (relative criteria), and dichotomized participants into responders and non-responders (46). Odds ratios (OR) with confidence limits were calculated by exact methods (StatXact) and interpreted by the scale of Hopkins (47). Effect size (ES) for the HOOS data were calculated as the fraction of median difference and IQR for the change scores of the total sample (48), in mathematical form [(Δ_E_ − Δ_C_)/IQR_pooled_], where Δ = change score, E = experimental group, C = control group and IQR = interquartile range. These ESs were interpreted according to the scale of Cohen (49): trivial (<0.2), small (≥0.2 < 0.5), moderate (≥0.5 < 0.8) or large (≥0.8).

For all analyses, statistical significance was considered at a two-tailed level ≤5%. The calculations were done on a personal computer using SPSS 12.0® (SPSS Incorporated), Excel 2002® (Microsoft Corporation) and Statxact 5® (CYTEL Software).

### Ethics

The study protocol was recommended by the Regional Committee for Medical Research Ethics, Western Norway (study no. 218.03) and approved by the Norwegian Social Science Data Service. It was conducted in accordance with the Declaration of Helsinki of 1975, as amended in 2000. Written and oral informed consents were obtained from all patients before inclusion.

## Results

### Subject characteristics

Small differences were seen at baseline between the two groups (Table I). However, the experimental group had more females, more bilateral hip pain, longer duration of pain and further distal pain irradiating.

The median, IQR and range of treatment sessions accomplished were 13.5, 5, 7–16 and 20, 6, 13–24 for the experiment and control groups, respectively (*p* = 0.007). There was negligible difference between groups in number of participants using analgesics and NSAIDs, both at baseline and follow-up (results not shown).

### Outcomes

At follow-up, all participants in the experimental group reported reduced disability in total HOOS compared with baseline (Table II). In the control group, four participants expressed deterioration and five improvements.

**Table II:** Raw scores in total HOOS for individual participants (P), given in medians at baseline (week 0) and follow-up (week 12).

	Experimental group (*n* = 10)				Control group (*n* = 9)		
			
P	Baseline	Follow-up	Change	P	Baseline	Follow-up	Change
E1	43	14	−29	C1	73	58	−15
E2	65	58	−7	C2	38	36	−2
E3	23	11	−12	C3	65	63	−2
E4	48	26	−22	C4*	45	37	−8
E5	63	36	−27	C5	53	34	−19
E6	36	31	−5	C6	42	52	10
E7	43	19	−24	C7	60	64	4
E8	56	34	−22	C8	35	44	9
E9	56	52	−4	C9	60	69	9
E10	48	14	−34				

The scale is graded 0–100, best to worst. Negative change scores express improvement.

* The participant given the group median change score. There are nine participants analyzed in the control group because the follow-up value for the one who dropped out was filled in by the analyzer according to the intention-to-treat principle. Total HOOS, Hip disability and Osteoarthritis Outcome Score HOOS = [(Σ5HOOS subscores)/5]; E1, experimental group participant one; C1, control group participant one.

In the total HOOS, there was a statistically significant difference in favour of the experimental group. This difference was also seen in three of five subscales (Table III). The within-group difference in total HOOS was a 43% and 3% improvement in the experiment and the control groups, respectively (results not shown). In Pain, the same figures were 63% and 25%, respectively.

**Table III:** Between-group comparisons in total HOOSt and in subscales.

Variables	Group	Baseline	Follow-up	BGD (CI)	*p*-values
HOOSt	E	48 (17)	29 (26)		
	C	53 (23)	48 (26)	−20 (−6, −31)	0.001*
Stiffness	E	43 (21)	25 (24)		
	C	55 (25)	55 (30)	−15 (−6, −25)	0.005*
Pain	E	46 (28)	17 (14)		
	C	44 (19)	33 (13)	−18 (−6, −32)	0.067
ADL	E	38 (28)	19 (33)		
	C	41 (18)	37 (22)	−21 (−2, −21)	0.045*
R&S	E	63 (31)	25 (39)		
	C	56 (22)	59 (25)	−31 (−15, −50)	0.045*
HR-QL	E	59 (16)	47 (31)		
	C	63 (54)	63 (30)	−13 (6, −25)	0.24

Absolute values are given in medians and interquartile range. The experimental group (*n* = 10) and the control group (*n* = 9). Baseline test (week 0), follow-up test (week 12), BGD, between-group difference; CI, 95% confidence interval; E, experiment; C, control. Significance tested by Mann–Whitney *U*-test. HOOSt, median total Hip disability and Osteoarthritis Outcome Score [(Σ5HOOS subscores)/5]; Stiffness, Symptoms others including stiffness; ADL, Activity limitation in daily living; R&S, Activity limitation in recreation and sport; HR-QL, Hip-related quality of life.

* Statistical significant difference (α≤50.05). Scores: 0 (no disability)–100 (worst possible disability).

Judged by the absolute criterion in the main outcome total HOOS, more patients responded in the experimental group (six of 10) than in the control group (none of 9), a difference that was statistically significant (Table IV). Also by the relative criterion in the total HOOS, more patients responded in the experimental than in the control group, but the difference was not statistically significant.

**Table IV:** Participants dichotomized into responders and non-responders according to scores on total HOOS and its subscales.

	t-HOOS	Stiffness	Pain	ADL	R&S	HR-QL
Improvement ≥20 points						
No responders (E, C)	6, 0	4, 0	7, 2	3, 0	7, 2	1, 2
*p*-values	0.002*	0.087	0.07	0.211	0.07	0.058
Odds ratio	inf	–	8.2	–	8.2	0.39
(95% CI)	1,6-inf†		0.75–113		0.75–113	0.006–9.4
Improvement ≥50%						
No of responders (E, C)	4, 0	5, 0	8, 1	5, 1	6, 1	0, 0
*p*-values	0.087	0.057	0.005*	0.141	0.057	–
Odds ratio	–	–	32.0	8.0	12.0	–
(95% CI)			1.8–1590	0.56–425	0.83–619	

Experimental (E) group (*n* = 10) and control (C) group (*n* = 9), tested for statistical significance by Fisher's exact test.

† The exact lower confidence limit for odds ratio. Inf, infinity; t-HOOS, the total Hip disability and Osteoarthritis Outcome Score = [(Σ5HOOS subscores)/5], Scores: 0 (no disability)–100 (worst possible disability); Stiffness, Symptoms others including stiffness; ADL, Activity limitation in daily living; R&S, Activity limitation in recreation and sport; HR-QL, Hip-related quality of life.

* Statistically significant difference (α≤55%). CI, confidence interval.

In subscales, by both absolute and relative criteria, more participants responded in the experimental than the control group in all but Hip-related quality of life (HR-QL) (Table IV). Only in Pain was the difference statistically significant. The number of patients responding in HR-QL by the absolute criterion was higher in the control group, whereas by the relative criterion there was equality amongst the groups. The effect magnitude in Pain by OR was very large by the relative criterion (Table IV).

All ESs were in favour of the experimental group (Table V). The ESs were large in total HOOS, as well as in the subscales Symptoms others including stiffness (Stiffness), Activity limitation in daily living (ADL), and Activity limitation in recreation and sport (R&S). In the subscales Pain and HR-QL, the ESs were moderate.

**Table V:** Effect sizes for total HOOS and its subscales.

	Total HOOS	Stiffness	Pain	ADL	R&S	HR-QL
Pooled variability	21	15	31	15	34	25
Effect size	1.1	1.0	0.7	0.8	1.0	0.6

Pooled variability of both experimental and control group. HOOS, Hip disability and Osteoarthritis Outcome Score HOOS = [(Σ5HOOS subscores)/5]; Stiffness, Symptoms others including stiffness; ADL, Activity limitation in daily living; R&S, Activity limitation in recreation and sport; HR-QL, Hip-related quality of life. Confidence intervals lacking due to non-parametric statistical tests.

## Discussion

The participants with hip disability who received a compiled physiotherapy program including graded traction mobilization up to 800 N reported statistically significant and superior important clinical effects in total HOOS and Pain after 12 weeks compared with the control participants who received a compiled physiotherapy program including unknown traction forces. The number of treatments used in the forceful mobilization group was 33% less then in the standard treatment group.

The therapists performed fewer treatments over a shorter period than stated in the protocol. They explained this as a deliberate judgment related to assessed normalized accessory hip motion, and the patient's describing satisfactory symptom reduction. The therapists seem to have violated the study protocol in the best interest of their patients. If the trial protocol had criteria for ending treatment as patients reduced symptoms under a certain limit, the ethical standard of the trial might have been improved. This would have required follow-up tests with closer intervals, which might had given data regarding the onset of the experimental treatment effect. However, it is plausible that the differences in outcome might have been even larger if the therapists had strictly complied with the trial protocol, as it is unknown if the effect of forceful traction follows an increase in capsular elasticity. Change in symptoms and accessory motion due to traction mobilization alone is to be scrutinized.

This trial presents only short-term results. The long-term effect ought to be assessed, considering the large ESs seen. We suggest that treatment effects of all the modalities in the experimental group should be further scrutinized.

The small number of participants in the final trial makes it necessary to interpret the results with caution. The *a priori* set power of 80% required a standardized treatment difference of 80% for the primary outcome. However, the larger treatment difference actually seen still afforded adequate power for total HOOS and Pain.

There might be several reasons why only 19 patients were enrolled in the inclusion period. Simultaneously to this trial, two other research groups were recruiting patients with nearly identical inclusion criteria in Oslo County. In addition, all patients with hip OA in Norway are entitled to physiotherapy free of charge from our National Health Service. Several physiotherapists stated frankly that treating these patients was a source of steady income. By participating in the trail, they would risk losing them to another clinic. We hypothesize economic and strategic reasons also to have influenced the general practitioners, as they tend to ignore evidence-based non-medical treatment for hip OA patients (50). KV visited 24 of the largest clinics, in both medicine and physiotherapy, presenting the trial and its importance. Nevertheless, no more than four physiotherapy clinics would commit to letting us mail information to patients on their waiting list.

Force difference in traction mobilization is not the only cause of the effect in this trial. There was suboptimal control regarding the additional treatment performed in the two groups. One experiment therapist has developed specialized exercises – both for strengthening and stretching – targeting the small external rotators of the hip in particular (33,34). Both experimental therapists worked by his principles. The result then might as likely be caused by these exercises as by the forceful mobilizations. On the other hand, it might seem unlikely that these exercises should be twice as effective as exercises used in other trials (51). In addition, testing the effect of mobilization without other treatment modalities might be invalid, as manual therapy is seen as only one remedial action of several building the total care (52). Still, our hypothesis is that forceful mobilization has important clinical value for hip disability even as a single modality.

The therapists performing the experimental treatment might have been better craftsmen than those in the control group. If the same therapists were to treat both groups, these personal factors could have been better controlled. On the other hand, this might have affected the therapist's belief in the treatment, and thus lowered the placebo effect in the control group. This might also have led to (patient) biasing intergroup contacts. As forceful traction mobilization feels quite different from low-force mobilization, the result might have been a lowered placebo effect in the control group related to decreased therapists' and patients' expectations.

The reliability of force application might have been more closely examined. However, serious efforts were made to standardize the force application in the experimental group. The therapists used clinic-like procedures regarding accuracy and time efforts in both groups, which made the interventions easily applicable in a private practice setting, enhanced the replication of the trial, and increased the generalization of the result. Imposing extra effort on the control therapists made it harder to recruit patients. This is the reason the force used in the control group was unknown, i.e. it reflects standard practice.

In this study, only the data puncher and the ROM raters were blinded. The trial administrator, KV, was blinded to all but the last patient in each randomization block, but should have been totally blinded. Not blinding the patients made them prone to bias, by their own and therapists' outcome expectations. Still, according to the scale afforded by Jadad et al. (53), we rated the trial quality to be 3/5 points.

The present data has raised suspicion against the responsiveness of one of the items in the subscale HR-QL: “How often do you think about your hip?”, since only one out of 19 participants reported to have changed their frequency of thinking. Maybe the question rather should be: “How often do you have negative thoughts about your hip?” The young HOOS scale needs further testing (36).

The main outcome measure might rather have been function and pain, as recommended by the Osteoarthritis Research Society International for OA clinical trials (46). This would have facilitated the direct comparison with earlier trials. Still, the multi-factor total HOOS score might give even a more informative picture of the health problems experienced by these patients.

The HOOS is an ordinal scale. Statistical methods for data from rating scales is said to differ completely from traditional methods for quantitative variables, since calculations based on adding or subtracting ordinal data are not appropriate (54). On the other hand, others find that parametric methods can also be used for ranked data (55). We choose to calculate median change scores even though the data are on an ordinal scale level.

We unwarily used a different method for calculating the total HOOS scores from the one prior validated. The validated method is to sum all the raw scores, divide them by 5 and then multiply by 100. The effect of not using this method is that the subscales with fewer items gain more weight on HOOS. Stiffness and HR-QL are the subscales with the fewest items, and, as the ESs were large and moderate in Stiffness and HR-QL, respectively, this might not be seen as a threat to the validity of our calculations. This was supported by our recalculation of total HOOS. Our re-analysis showed the same statistically significant between-group difference as before.

This study is the first RCT to show clinically important statistically significant treatment outcomes related to a compiled physiotherapy program including forceful manual traction mobilization in patients with hip disability. This supports the hypothesis of inadequate use of force progression in prior mobilization trials (16,17).

The subjects included displayed equality on most baseline factors, which is considered to strengthen the results (25). The ES seen in this trial is 2–4 times larger than reported in hip OA information trials (28) and exercise trials (56). Hence, this might support the hypothesized causation of the forceful traction mobilizations. Notwithstanding, the effect of the compiled approach is consistent.

The manual traction mobilizations method of Samuelsen & Høiseth (21) might be regarded a highly effort-demanding approach. However, in support of the feasibility of the method, normally strong female 50-year-old physiotherapists are fully capable of handling this force, as documented by measurements taken with our hanging scale arrangement.

To take out the 1–1.5 cm of accessory motion of the hip by traction, forces amounting at least 400–600 N are probably required (21,22). Prior to the trial, we made an experiment using the technique applied in the control group (23), which showed the bench moving forward on the floor before the scale showed 350 N. This force was applied to a model of a foot, tied to a hanging scale, again fixed to a regular heavy therapy bench with a person weighting 770 N on top. The bench was placed on wood with a floor sealer and floor covering, but the results were similar. It therefore seems that either external or therapist fixation of the plinth is a presupposition for effective hip mobilizations.

A recent review on exercise treatment for hip OA (51) included only two high-quality studies. Both studies reported small to moderate ESs regarding pain and function, respectively. According to the much larger ESs seen in the present study, exercise therapy is suggested only as a supplement to forceful traction mobilization treatment and manipulations (14) in patients with hip disability.

For improving function and ROM in even more hypomobile patients than in this trial, like those reported in the study of Hoeksma et al. (14), forceful abrupt traction and rotational manipulations might be the first line of treatment. This is derived from the fact that our most reliable ROM measures (43) showed minimal change after treatment, whereas Hoeksma et al. (14) reported great increase in ROM in most participants. Nevertheless, our measurements were unreliable, and we also have to question data reported from other researchers (13,14), as they did not report absolute reliability values. In sum, knowledge about the effect of both forceful mobilization and manipulation on ROM is uncertain. The frequency and force needed to achieve hip joint hysteresis by traction mobilization warrants further investigations. The fact that two raters, even though they were still students, were not able to reliably measure ROM, makes it reasonable to question also the reliability of KV's estimates when testing the participants for eligibility. In future trials, cinematographic evaluation of hip ROM might be needed to accurately secure the hypomobility criteria (57). By such technology, it should be possible to answer the effect of manual physiotherapy interventions on ROM in these patients.

NSAIDs are highly recommended by general practitioners as a treatment for lower limb OA (58), even though there are few studies of adequate quality giving explicit data regarding treatment in hip OA (58–61), and the extracted ESs regarding pain and function are reported to be small (0.2–0.3) compared with placebo treatment (58). The present trial showed 3–4 times larger ESs than this and even had an active treatment comparison group. Taking into account the seriously adverse effects seen in short-term drug studies (60,62), forceful traction mobilization and manipulations might be better choices of treatment in patients with hip OA or hip disability.

## Conclusion

The findings suggest clinically important post-treatment effects by a compiled physiotherapy program including forceful traction mobilizations offered by more hip specialized therapists in patients with hip disability in primary healthcare. The long-term effect is not known. Scientists might seek the normal variation of *in vivo* hip capsular stiffness in healthy adults. This is a presupposition for determining whether therapists are truly able to differentiate between people with normal versus hyper hip stiffness, and if forceful traction mobilization can actually reduce hip stiffness. They might also seek for the force, frequency, and volume of elongation and relaxation cycles needed to achieve the hysteresis effect. Such data might be a basis for RCTs with larger sample size, further blinding, valid ROM measurements, known forces in both groups, standardized information and exercise regimes, more frequent follow-ups, and longer follow-up periods. Then, in the future, we might know more about which part of the treatment protocol is causing the effect, the effects onset time and the true ES. The experimental approach seems promising and has shown no side-effects in this trial.

## References

[1] WHO (2001). ICF – International classification of functioning, disability and health.

[2] Sunden-Lundius A, Johnsson B, Lohmander S, Ekdahl C (2005). Prevalence of self-reported hip disorders, relations to age, gender, pain, stiffness, weakness and other joint disorders. Adv Physiother.

[3] Danielsson L, Lindberg H (1997). Prevalence of coxarthrosis in an urban population during four decades. Clin Orthop Relat Res.

[4] Picavet HS, Hazes JM (2003). Prevalence of self reported musculoskeletal diseases is high. Ann Rheum Dis.

[5] Birrell F, Croft P, Cooper C, Hosie G, Macfarlane G, Silman A (2000). Health impact of pain in the hip region with and without radiographic evidence of osteoarthritis: A study of new attenders to primary care. The PCR Hip Study Group. Ann Rheum Dis.

[6] Altman RD (1995). The classification of osteoarthritis. J Rheumatol Suppl.

[7] Altman R, Alarcon G, Appelrouth D, Bloch D, Borenstein D, Brandt K (1991). The American College of Rheumatology criteria for the classification and reporting of osteoarthritis of the hip. Arthritis Rheum.

[8] Bierma-Zeinstra S, Bohnen A, Ginai A, Prins A, Verhaar J (1999). Validity of American College of Rheumatology criteria for diagnosing hip osteoarthritis in primary care research. J Rheumatol.

[9] Klassbo M, Harms-Ringdahl K, Larsson G (2003). Examination of passive ROM and capsular patterns in the hip. Physiother Res Int.

[10] van Baar ME, Dekker J, Oostendorp RA, Bijl D, Voorn TB, Bijlsma JW (2001). Effectiveness of exercise in patients with osteoarthritis of hip or knee: Nine months' follow up. Ann Rheum Dis.

[11] Kettunen JA, Kujala UM (2004). Exercise therapy for people with rheumatoid arthritis and osteoarthritis. Scand J Med Sci Sports.

[12] van Baar ME, Assendelft WJ, Dekker J, Oostendorp RA, Bijlsma JW (1999). Effectiveness of exercise therapy in patients with osteoarthritis of the hip or knee: A systematic review of randomized clinical trials. Arthritis Rheum.

[13] van Baar ME, Dekker J, Oostendorp RA, Bijl D, Voorn TB, Lemmens JA (1998). The effectiveness of exercise therapy in patients with osteoarthritis of the hip or knee: A randomized clinical trial. J Rheumatol.

[14] Hoeksma HL, Dekker J, Ronday HK, Heering A, van der Lubbe N, Vel C (2004). Comparison of manual therapy and exercise therapy in osteoarthritis of the hip: A randomized clinical trial. Arthritis Rheum.

[15] Maitland GD (1991). Peripheral manipulation.

[16] Nyfos L (1983). Traction therapy of osteoarthrosis of the hip. A controlled study. Ugeskr Laeger.

[17] Marques B, Toldbod M, Ostrup EL, Bentzen L, Gjorup T, Gylding-Sabroe JP (1983). The effect of naproxen compared with that of traction in patients with osteoarthrosis of the hip. A single-blind controlled study. Ugeskr Laeger.

[18] Weber VE (1974). The use of manual traction as conservative treatment in hip osteoarthritis. Beitr Orthop.

[19] Angstrom L, Lindstrom B (2003). Treatment effects of traction and mobilization of the hip joint in patients with inflammatory rheumatic diagnoses and hip osteorarthritis. Nordisk Fysioterapi.

[20] Kaltenborn F (1980). Mobilization of the extremity joints: Examination and basic treatment techniques.

[21] Samuelsen G, Høiseth A (1990). X-ray examination: Manual physical traction treatment of painful hip conditions. Fysioterapeuten.

[22] Arvidsson I (1990). The hip joint: Forces needed for distraction and appearance of the vacuum phenomenon. Scand J Rehabil Med.

[23] Kaltenborn F (2002). Manual mobilization of the joints: The Kalten-born method of joint examination and treatment; volume I. The extremities.

[24] Swinscow TDV (2002). Statistics at square one.

[25] Altman DG (1991). Practical statistics for medical research.

[26] Staff PH, Kvien TK (1988). Arthroses. Guidelines for diagnosis and treatment. Tidsskr Nor Laegeforen.

[27] Roach KE, Miles TP (1991). Normal hip and knee active range of motion: The relationship to age. Phys Ther.

[28] Klassbo M, Larsson G, Harms-Ringdahl K (2003). Promising outcome of a hip school for patients with hip dysfunction. Arthritis Rheum.

[29] Hesch J, Vleeming A, Mooney V, Dorman T, Snijders C, Stoeckart R (1997). Evaluation and treatment of the most common patterns of sacroiliac joint dysfunction. Movement stability and low back pain – The essential role of the pelvis.

[30] Butler DS (2000). The sensitive nervous system.

[31] McKenzie R, May S (2003). The lumbar spine mechanical diagnosis and therapy I.

[32] Greenman PE (1996). Principles of manual medicine.

[33] Evjenth O, Hamberg J (1989). Autostretching: The complete manual of specific stretching.

[34] Samuelsen G, Juel NG (1997). Quadriceps coxae – A biomechanical analysis.

[35] Samuelsen G, Hoiseth A, Lillas F, Dahl HA (1996). The external rotators of the hip – Underestimated and misunderstood?. Fysioterapeuten.

[36] Klassbo M, Larsson E, Mannevik E (2003). Hip disability and Osteoarthritis Outcome Score. An extension of the Western Ontario and McMaster Universities Osteoarthritis Index. Scand J Rheumatol.

[37] Nilsdotter AK, Lohmander LS, Klassbo M, Roos EM (2003). Hip disability and Osteoarthritis Outcome Score (HOOS) – Validity and responsiveness in total hip replacement. BMC Musculoskelet Disord.

[38] Sartorius N, Kuyken W, Orley J, Kuyken W (1994). Translation of health status instruments. Quality of life assessment: International perspectives.

[39] Klassbo M (2003). Hip disability – Patient education, classification and assessment.

[40] Vaarbakken K (2005). The hip disability and osteoarthritis score (HOOS) version LK1.1. www.liv.se/fof.

[41] Holm I, Bolstad B, Lutken T, Ervik A, Rokkum M, Steen H (2000). Reliability of goniometric measurements and visual estimates of hip ROM in patients with osteoarthrosis. Physiother Res Int.

[42] Norkin CC, White DJ (1995). Measurement of joint motion: A guide to goniometry.

[43] Vaarbakken K (2005). Physiotherapy for painful and hypomobile hip joints – Theoretical considerations an a trial comparing standard to forceful manual traction mobilization.

[44] Hollis S, Campbell F (1999). What is meant by intention to treat analysis? Survey of published randomised controlled trials. BMJ.

[45] Campbell MJ, Gardner MJ (1988). Calculating confidence intervals for some non-parametric analyses. BMJ (Clin Res Ed).

[46] Pham T, van der Heijde D, Altman RD, Anderson JJ, Bellamy N, Hochberg M (2004). OMERACT-OARSI initiative: Osteoarthritis Research Society International set of responder criteria for osteoarthritis clinical trials revisited. Osteoarthritis Cartilage.

[47] Hopkins WG (1997). A new view of statistics: Effect magnitudes. Monograph on the Internet. www.sportsci.org/resource/stats/effectmag.html.

[48] Kazis LE, Anderson JJ, Meenan RF (1989). Effect sizes for interpreting changes in health status. Med Care.

[49] Cohen J (1988). Statistical power analysis for the behavioral sciences.

[50] Bierma-Zeinstra SM, Lipschart S, Njoo KH, Bernsen R, Verhaar J, Prins A (2000). How do general practitioners manage hip problems in adults?. Scand J Prim Health Care.

[51] Fransen M, McConnell S, Bell M (2003). Exercise for osteoarthritis of the hip or knee. Cochrane Database Syst Rev.

[52] Moritz U, Johanssen B (1992). Manipulation of the spine is only a part of the total care. Lakertidningen.

[53] Jadad AR, Moore RA, Carroll D, Jenkinson C, Reynolds DJ, Gavaghan DJ (1996). Assessing the quality of reports of randomized clinical trials: Is blinding necessary?. Control Clin Trials.

[54] Svensson E (2001). Guidelines to statistical evaluation of data from rating scales and questionnaires. J Rehabil Med.

[55] Scheff SW, Saucier DA, Cain ME (2002). A statistical method for analyzing rating scale data: The BBB locomotor score. J Neurotrauma.

[56] van Baar ME, Assendelft WJ, Dekker J, Oostendorp RA, Bijlsma JW (1999). Effectiveness of exercise therapy in patients with osteoarthritis of the hip or knee: A systematic review of randomized clinical trials. Arthritis Rheum.

[57] Neumann DA (2002). Kinesiology of the musculoskeletal system: Foundations for physical rehabilitation.

[58] Bjordal JM, Ljunggren AE, Klovning A, Slordal L (2004). Nonsteroidal anti-inflammatory drugs, including cyclo-oxygenase-2 inhibitors, in osteoarthritic knee pain: Meta-analysis of randomised placebo controlled trials. BMJ.

[59] Towheed T, Shea B, Wells G, Hochberg M (2000). Analgesia and non-aspirin, non-steroidal anti-inflammatory drugs for osteoarthritis of the hip. Cochrane Database Syst Rev.

[60] Lee C, Straus WL, Balshaw R (2004). A comparison of the efficacy and safety of nonsteroidal antiinflammatory agents versus acetaminophen in the treatment of osteoarthritis: A meta-analysis. Arthritis Rheum.

[61] Zhang W, Doherty M, Arden N, Bannwarth B, Bijlsma J, Gunther KP (2005). EULAR evidence based recommendations for the management of hip osteoarthritis: Report of a task force of the EULAR Standing Committee for International Clinical Studies Including Therapeutics (ESCISIT). Ann Rheum Dis.

[62] Singh G, Wu O, Langhorne P, Madhok R (2006). Risk of acute myocardial infarction with nonselective non-steroidal anti-inflammatory drugs: A meta-analysis. Arthritis Res Ther.

